# “People are shortening the lifetime of mentally ill persons”; Community’s perception towards mental illness and help-seeking behavior in Bench Sheko, Sheka, Kaffa and West Omo zones, South West Ethiopia, 2021

**DOI:** 10.1371/journal.pone.0320740

**Published:** 2025-04-29

**Authors:** Nahom Solomon Habtamu, Gebremeskel Mesafint, Kidus Yenealem, Sewagegn Demelash, Zenebu Muche, Dawit Getachew

**Affiliations:** 1 Department of Public Health, School of Public Health, Mizan-Tepi University, Mizan, Ethiopia; 2 Department of Psychiatry, School of Medicine, Mizan-Tepi University, Mizan, South West Ethiopia; 3 Department of Social Work, Mizan-Tepi University, Mizan, South West Ethiopia; 4 Department of Psychology, Debre-Markos University, Debre Markos, East Gojam, Ethiopia; Caleb University, NIGERIA

## Abstract

**Background:**

Mental diseases or mental disorders are conditions that affect the mind and are defined by changes in emotion, thought, or behavior. Many studies have demonstrated that people with mental illnesses are not receiving the necessary care and treatments, despite the fact that they need basic healthcare services and supportive strategies. Particularly in Ethiopia, there is a severe paucity of research on the topic. The aim of this study was to investigate how South West Ethiopians perceive and act when seeking treatment for mental illness.

**Methods:**

We conducted a descriptive qualitative study in Bench-Sheko, Kaffa, West Omo, and Sheka Zones, Southwest Ethiopia, from February to May 30, 2021. A purposively selected 58 individuals were interviewed by research experts using interview guides and responses were audio recorded. The recorded data were transcribed verbatim and analyzed by following a thematic analysis approach and the findings were narrated by supporting with quotes.

**Results:**

In this study, 58 participants took part, and they were let to discuss on: the definitions of mental disease, its origins and symptoms, caregiving practices, methods of treatment, and difficult tasks related to mental illness. The study showed that people’s perceptions and descriptions of people with mental illnesses, as well as how they interact with and behave when seeking care, were discriminatory. In the early stages of the illness, some family members attempt to take the mentally ill individuals to their respective places of worship, but very rarely to medical facilities. However, as the illness progresses, almost all mentally ill individuals are typically left to live on the streets, where they frequently encounter prejudice and social exclusion.

**Conclusion:**

The local community does not take a supportive stance toward those who are mentally ill, and there is no formalized system in place for them to receive care and treatment. Few people who are willing to give them food, clothing, and shelter were proven to have the power to influence their fate. Therefore, it is crucial to provide mental health services to those in need and develop effective awareness-raising initiatives, especially by working with local authorities, religious leaders, and other influential individuals.

## Introduction

Mental health is a state of well-being in which an individual can realize his or her own abilities, interact positively with others, cope with the stressors of life and work productively, and contribute to his or her family and community [1]. It is a key determinant of overall health and socio-economic development. It influences a variety of outcomes for individuals and communities [2,3]. On the other hand, mental illnesses or mental disorders are health conditions involving changes in emotion, thinking, or behavior. They are associated with distress and/or problems functioning in social, work, or family activities. There are many kinds of mental disorders mainly including depression, bipolar disorder, schizophrenia and other psychoses, dementia, and developmental disorders including autism [2,4,5].

Across the globe, different factors are reported to be causes of mental illness including; hereditary, negative life events, brain disease and medical problems, God’s punishment, personal weakness [6] Substance use, poverty, family problems [7], bewitch, accident, unemployment, and curse [8,9]. On the other hand, there were study reports that show Poor knowledge [8,10–13], attitude, and perceptions regarding mental illness [8,12–15]. There is also a report that revealed negative perceptions regarding approaching someone with a mental disorder, encouraging doubt and fear [16].

Scholars have put in place different effective strategies for preventing and treating mental disorders and ways to alleviate the suffering caused by them. Most commonly used treatment options include; mental hospital (main), counseling, traditional healers [8,17], faith healers [12,15,18], and family support [8]. Again it is recognized that all persons with mental illness will enjoy the full range of human rights on an equal basis with others and no one will be discriminated on the grounds of mental illness and disability [19]. However, misperception which results in Stigma, discrimination, and human rights abuses are part of the daily lived experience of the mentally ill people and even their families [20–22].

Many are denied economic, social, and cultural rights, with restrictions on the rights to work and education as well as reproductive rights and the right to the highest attainable standard of health. They may also be subjected to unhygienic and inhumane living conditions, physical and sexual abuse, neglect, as well as harmful and degrading treatment practices in health facilities [3]. On the other hand, there is a belief that the best way to handle mentally ill patients is to keep them behind locked doors, and mentally ill patients are a burden on society, and it is best to avoid anyone who has mental problems. Similarly, describing it as shameful to mention someone in the family who has a mental illness [23].

In Ethiopia, mental illness is the leading non-communicable disorder in terms of burden. Indeed, in a predominantly rural area of Ethiopia, mental illness comprised 11% of the total burden of disease, with schizophrenia and depression included in the top ten most burdensome conditions, and despite the existence of affordable and effective treatments, fewer than one in 10 of the most severely affected people ever receive the treatment they need. The help-seeking behavior and experience is also very low in the country. People give different meanings to mental illness and mention multiple things to be a cause of mental illness [20].

The most common word used to describe or name a mentally ill person by Ethiopians is madness or mad (Ibd-in Amharic language). Other names used to describe a person assumed to have mental illness include; in the Amharic language, “cherqun yetale” which denotes a severely mentally ill person unfit for any responsibility or “Wofeffe” or “nik” which also implies that a, person is not too reliable because he/she has inconsistent behavior due to episodic explosiveness or other unusual changes in his/her inter-personal communication [24].

Regarding the cause and treatment of mental illness; it is common for Ethiopians to associate it with evil spirits and things related to supernatural power. Other assumed causes include; stress and life complexities, *Buda (evil eye), magicians’ power, Danqara/Metet, Poisoning, alcohol, chat* [24,25]*.* For treating mentally ill people, Ethiopians mostly use traditional and religious healing methods [24].

There are gaps in understanding mental illness from its causes to treatment approaches which highly demand attention. For that; having strong scientific evidences are very important [20,26]. We have learned that mental illness is not well studied in Southwest Ethiopia where many diversified people are living. This study has explored the communities’ perception toward mental illness in four zones of South West Ethiopia and we believe it will be helpful to work on mental health in the given area to other similar settings.

## Methods

### Study area

This study was conducted in Bench-Sheko, Kaffa, West Omo and Sheka Zone, Southwest Ethiopia. The information gathered from respective Zonal Health Bureaus indicated that;-

Kaffa zone, with its capital of Bonga is located at a distance of around 460 km from Addis Ababa. The zone has 12 districts and 2 town administrations. It has a total of 1,196,565 populations with 586,317 males and 610,248 females. As the zone’s health bureau report shows; there are 3 hospitals (1 general and 2 primary hospitals), 43 health centers and 293 health posts, of which 272 are functional by the year 2018. The zone has a total of 843 health professionals at hospital and health center levels and 524 at health posts level.

Bench Sheko Zone, with its capital of Mizan Aman town is located around 585km away from Addis Ababa. The zone has two town administrations and 6 rural districts with total of 625,345 residents, 1 Teaching Hospital, 26 Health centers with 904 Health professionals, and 224 Health posts with 567 Health professionals.

Sheka zone is located in SNNPR, around 700 km from the capital city, Addis Ababa, with its center, Masha and Tepi town. There are three districts and two town administrations. As the zonal health bureau report indicates; it has one functional hospital with 10 general practitioners and other 135 health professionals. Again there are a total of 227 diploma and degree holder health professionals working at different public health centers and 111 health extension workers in the zone.

West Omo Zone is part of the South West Ethiopia with its capital town called Jemu which is about 700 km away from Addis Ababa. It is a newly formed zone that has different districts with multiple languages and cultures. The communities were Urban, semi-urban to rural and pastoral communities. The zone has 2 district hospitals, 13 Health centers, and 96 Health posts.

### Study design and period

A descriptive qualitative study approach, which helps to get details of the study issue was conducted from 1st February of 2021 to May 30, 2021. Descriptive qualitative research is known by its naturalistic and holistic features involving a rich collection of data from various sources to gain a deeper understanding of individual participants, including their opinions, perspectives, and attitudes [27,28].

### Population

#### Source population.

The **source population** of this study were all adult population aged 18 years and above residing in Bench-Sheko, Kaffa, West Omo and Sheka Zone for the last six months prior to the data collection period.

#### Study population.

All adult population aged 18 years and above residing in Bench-Sheko, Kaffa, West Omo and Sheka Zone for the last six months and who fulfill the eligibility criteria in the selected kebeles.

### Inclusion and exclusion criteria

#### Inclusion criteria.

All adult population aged 18 years and above residing in Bench-Sheko, Kaffa, West Omo and Sheka Zone for the last six months prior to the data collection period.

#### Exclusion criteria.

People who were sick and unable to properly communicate during the data collection period were not included in the study.

### Sample

The study has involved study participants from all zones mainly focusing on persons who are assumed to have detailed information about mental illness. Accordingly; a total of 58 people who are community members, community leaders, religious leaders, social workers, health professionals and government bodies were involved as in-depth and key informant interview participants. The number of study participants was limited to 58 because of the saturation of the information at that level.

### Data collection procedures

An interview guide for both in-depth interview and key informant interview participants was prepared based on the objective and the interview was conducted by trained health professionals. The key points in the interview guide were regarding the meaning/naming of mental illness and mentally ill people, perception towards mental illness, how could mental illness occurs?, what care and supports are there to mentally ill people and how is the help-seeking behavior?

### Ensuring trustworthiness and data quality

Two days of training was given for data collectors and supervisors regarding the objectives of the study, data collection method, and significance of the study. During data collection, each data collector was supervised and daily meeting was conducted between data collectors, supervisors, and principal investigators for discussion and to assess the progress of data collection. As far as trustworthiness is a basic issue in research we have applied the recommended procedures to secure the credibility, dependability, confirmability and transferability of this study. Accordingly, the data collectors had approached participants very friendly and developed rapport. Interviews were audio recorded and kept for cross checking as needed. Audit trial by research experts, debriefing and feedback from colleagues and supervisors were applied in managing the data for credibility. The inquire process and findings were described in detail so that anyone could learn about the details of the study for transferability. Documents of all the study process were kept and research experts were let to see the neutrality and dependability of the data. A code-recode process was done on separate time and checked for similarity of codes for intra and inter-coder dependability. Conformability was maintained by audit trail and participants’ words were presented as quotes in writing the findings.

### Data analysis

Data analysis involves organizing the data, conducting a preliminary read-through of the database, coding and organizing themes, representing the data, and forming an interpretation of them [29]. Data analysis for this study was done simultaneously with data collection. The information stored on the audio recorder was transcribed verbatim by the interviewers. Investigators developed a codebook and made discussions on the coding process. A thematic analysis was applied to summarize the findings. In doing that we passed through different levels of analysis including:- getting ourselves familiar with the collected data, generating initial codes, bringing initial codes into potential themes, reviewing and refining those themes derived from the initial codes, defining, naming, and organizing those themes. As it is known, thematic analysis gives an opportunity to understand the potential of any issue more widely and allows to determine the relationship between concepts and compare them with the replicated data. Again, thematic analysis helps to link the various concepts and opinions of the learners and compare these with the data that has been gathered in different situations at different times during the project. Furthermore; thematic Analysis could be appropriate when the study aims to understand the current practices of any individual. In particular the influence of any variable, which is utilized by participants in a practical way in order to investigate and identify how current situations are influenced by their points of view [30,31]. Having those concepts and applying all required processes, the full report of this study was produced and presented supporting it with quotes from participants’ responses.

### Ethical consideration

This study was conducted after passing through repeated proposal review and getting approval by the Institution Research Board of Mizan-Tepi University (with a letter of MTU/00120/21). Permission and supportive letter was also obtained from the Research and Community Service director of the college and health bureaus of the 4 zones and selected districts. Written informed consent was taken from study participants after interviewers explained details of the research objectives, purpose, risk, benefit, participants’ rights and confidentiality of the study. The study participants were informed as they have the right to withdraw from the study any time they need or skip questions, as there will not be direct benefit and no harm due to participating in the study except taking few minutes for answering the questions. The participants were also informed as the information they provide will be kept confidential and merely used for research purpose. Altogether; we confirm that all methods were performed in accordance with the relevant research ethics guidelines and regulations.

## Study results

This study has focused on exploring the perception of mental illness and help seeking behavior among community members and the specific discussion points were:- meanings of mental illness, the causes and symptoms of mental illness, experience of mental illness, cares and approaches to mental illness, management options and preference of treatment options, and demanding works regarding mental illness. Based on the responses gathered from the study participants; the findings are summarized under two themes of; perception to mental illness and help seeking behavior, which includes four sub categories including:- background of study participants, meanings of mental illness, causes of mental illness, help seeking and preferences of treatments, cares and sources of support to mentally ill people.

### Background of study participants

A total of 58 people (34 were males) have participated in this study. Most of the study participants were married (73.7%), and aged 31–40 years old (43.1%) (Table 1). Eighteen of them were key informants from health professionals, social affair workers, religious leaders and community leaders, and others were community members. Participants were from rural and urban parts of the 4 zones namely: Bench Sheko Zone, Sheka Zone, West Omo Zone and Kaffa Zone.

**Table 1:** Socio-demographic characteristics of study participants, Bench Sheko Zone, Sheka Zone, West Omo Zone and Kaffa Zone, 2021 (N=58).

Variables	Category	%
Sex	Male	58.62
	Female	41.38
Age	20-30	13.8
	31-40	43.1
	41-50	20.7
	>50	22.4
Marital status	Single	26.3
	Married	73.7
Religion	Orthodox	46.55
	Protestant	43.1
	Muslim	10.3
Ethnicity	Kaffa	27.6
	Bench	15.5
	Sheka	12
	Me’enit	13.8
	Amhara	20.7
	Others(Oromo, Guraghe, Tigre)	10.34
Education	No formal Education	17.2
	Grade 1–8	31
	Grade 9–12	13.8
	Diploma and above	37.9
Residence	Urban	51.7
	Rural	48.3

### Perception to mental illness

Under this category; how people define and what meaning do they give to mental illness, how mentally ill people are described and what manifestations do they have, what leads to mental illness and experience of mental illness are discussed in detail.

#### Meanings of mental illness.

All study participants have agreed that mental illness is a disease that leads to loss of consciousness and is manifested by different presentations among affected people. Mental illness is also described as a disease in which one person is unable to think properly as a healthy person and fails to control himself and in which a person may misbehave, annoy the community, and do unnecessary things that are uncommon and unacceptable by the community. Again; Mental illness is assumed to be very harmful, which isolates the person from social life and mentally ill persons don’t get support and care rather they move to towns, live on the street, and face different harms. The following statements were part of responses from the study participants:

- *“Mentally ill people may demonstrate uncommon things like; may not respect people, may misbehave, may lack courtesy and do anything they need without any fear. They may wear clothes or may not dress. They may not think for future or for others too. They may insult others and even may attack or bit others.” (a woman-IDI*)
- *“Mental illness is a disease in which people might lose consciousness or face fall down incidentally… The first thing is loss of consciousness and deviating from the community’s norm like living in the street, walking bare body and not acting as healthy person. They may walk here and there unreasonably, show odd character (mad), may misbehave and not agree with others.” (a woman-IDI)*

One study participant has shared his view regarding mental illness and reported that people who are assumed to be mentally ill may not be really ill but rather may have unique thoughts than ordinary people and that may need special attention.

- *“I think mostly mental illness is considered as insanity. I don’t take mental illness as insanity; a mentally ill I think is someone who views the world from a different perspective than the way we look at the world. I don’t consider all people with mental illness have a disease. I think a person with mental illness is someone who has a different thought and world that is completely different from the thought of a normal person.” (a man-IDI)*

Different names were mentioned to be used to describe mentally ill people at different places and the most commonly mentioned names include; mad (Ibid), unstable (tesh), unconscious (yenekele, aemrow yeteneka, nik). There are also more names in the local language and it was reported that such names have a negative impact on the view and approach towards mentally ill people. Here below are what the study participants mentioned to describe mentally ill people:-

- *“In our community usually we named as ‘Ebd’ ‘shote yalew’ there is a lot of naming and usually the communities insulting them. This will lead further illness or worsening of the illness. People in my surrounding insult them and sometimes might hurt physically even though they are ill.” (a man-IDI)*
- *“People call them insane, they don’t have any positive portrayal. Even the people with mental illness rather choose to stay there than recover and hear these names.” (a man-IDI)*

Sources of information about mental illness and related services are found to be very limited in the study area and most participants have witnessed that people are not well informed about mental illness and lack adequate information about what the illness is about, what to do about it, and where to find support. The following response indicates that:

- *“I think there are institutions in big towns that provide treatment for mentally ill people; I think there are few institutions in the country. If there were institutions they will teach the community. They may create awareness and the religious institutions can work with the government since most of the mentally ill can be found in religious centers. The religious institutions can build a center that treats the mentally ill other than holy water and that will improve the situation.” (a man-KII)*

#### Causes of mental illness.

There are multiple views regarding the causes and how mental illness occurs. Majorities of study participants have described that mental illness is a result of life complexities, stress, frustration, and substance use (chat, alcohol, and others). On the other hand; few participants have reported that mental illness is a disease which is associated with an evil spirit resulting from sin and God’s punishment (Ye Egziabher kuta or mergem), and very few have mentioned that mental illness might be hereditary and accidents or head injuries during birth might be a cause for mental illness.

- *“Sometimes mental illness is associated with evil spirit (Kalcha), especially in rural area. People thought that if children fail to worship or commit what is needed to their belief in relation to kalcha, as that of their families, they may face mental illness. Again also people don’t speak loudly; stress is also a cause of mental illness. Another evil spirit related issue is the so called likift and muart.” (a man-KII)*
- *“If head of a newly born child touches the land or if the child fails on the ground and its head touches the ground; we think that it may get mental illness.” (a woman-KII)*
- *“It might be transferred from family or he community might think that Bad spirits or the spirits of the devil are dwelling on him in relation to cult belief. When he brings new idea they consider that as a bad spirit. They also say that somebody did a Magic scroll on him. For example a student might go to school or university and working good they return insane and people say it is because somebody did a magic on them. so the causes can be a lot of things.” (a man-IDI)*

Study participants themselves were asked as “Do you think you may get mental illness?” and almost all replied “Who Knows his future?” and have reported that they may face it because of multiple reasons like; life complications, stress, trauma, bad spirits, and others.

### Help-seeking behavior and cares to mentally ill people

Under this category; issues related to; what cares and supports are being given to mentally ill people, how people approach them, what kinds of treatment options are there, from whom people need help, how people seek support and responses for are discussed in detail.

#### Cares and sources of support to mentally ill people.

Most study participants believe that mentally ill people should get attention and basic medical services, cares, and supports however they have reported that it is not common to see people who need treatment and care get what they have to get. What is common is once a person develops a mental illness; families and friends may try to keep at home or take to religious places for a short period and at the end leave him/her to live alone or push him/her to go outside to the street or to other places. For that reason, almost all mentally ill people are living on the street and feed themselves by begging. Very few participants have mentioned that some mentally ill people may get some food and cloth aid, usually leftover food and old clothes from volunteer people occasionally. Here below are parts of the responses from the study participants:-

- *“We are not seeing support to mentally ill people from the community. Usually attention is given to support poor people (economically lower class people) than mentally ill people both from the government and the community. The community don’t approach mentally ill people because of fearing attack or else.” (a man-KII)*
- *“There are a lot of people with mental illness in our community. They don’t access any type of treatment still they are wandering throughout the town and some of them escaped out from attending holy water. I think the family member doesn’t have money to bring to a health facility.” (a woman-IDI)*

Some study participants have discussed that the problem is not merely a lack of care or support to mentally ill people rather what makes it worse is there are conditions in which such people face abuse from community members which aggravates their illness. It was reported that some people may laugh at them, insult or call them insane, approach them for fun, and consider them as inhumane. Three of the study participants from different areas have shared the following points regarding this issue:-

- *“There is no tangible support rather people abuse mentally ill people by calling them mad, shouting and laughing on them…sometimes people try to tie and take for holy water or health center but most live on the street without getting care and even facing stigma.” (a man-KII)*
- *All I see is people trying to intensify their illness rather than helping to improve their situation. ….. There is a high chance, people with mental illness will recover to normal if people gathered them and cared for them and given them love, but we don’t see that. Rather we see people making them angry and adding illness to their disease and shortening their life time. I don’t think people who have the knowledge and gift to treat those people and save them are born yet….Sometimes loyal friends and relatives may help on taking them to a holy water or to a medical center. I don’t think others participate like that and I don’t see it.” (a man-IDI)*
- *“People see mental illness as very different and they see it as some kind of ghostly. They see it as a very different disease. They see it as something to do with spirit. They stigmatize against them and they don’t consider them as human.” (a woman-IDI)*

#### Help seeking and preferences of treatments.

Help seeking is about experiences in seeking and providing support to a mentally ill people. Accordingly, most study participants have reported as they themselves have very limited experience in helping mentally ill people and also didn’t see others doing that. Only very few have reported experience of supporting mentally ill people very rarely and incidentally. Of course, the mentally ill people may not be able to search how and where to get support and it is the family and the community who are supposed to approach them and find solutions to them. However, it is reported as it is usually missed and the responsibility falls on persons who are very generous and volunteers. As a result many of mentally ill people are found to be neglected and their health condition getting deteriorating from time to time rather than looking promising things to better outcome.

- *“In our locality there is a tendency to distant oneself from people with mental illness. They don’t get close to them or talk with them or give greetings. They are not treated; they are treated by their families only. Much or less I saw the family treating them.” (a man-IDI)*
- *“We are not close to them since they show behavior that is not normal, for example a normal person doesn’t defecate in front of other people in the open. When they do things we won’t do in the open the community thinks this is not normal and I do too. They do unique things and even when we walking the street if they come close to us we fear they might hit us and distance ourselves. The community segregates them because they show behavior that deviates from the expectation in the community, since you live with other people you should behave like them. That is how I think.” (a woman-KII)*

Regarding preferences of treatments and care to mentally ill people; most have suggested taking mentally ill people to their respective religious places and get prayer and other support. The reason why most have preferred religious places is because they believe the causes of mental illnesses are more of spiritual related and the solution is also from God. Again lack of healthcare facility that provide medical services to mentally ill people was also mentioned to be the reason to prefer religious places. Some others have revealed that taking mentally ill people to both religious places and healthcare facilities will be beneficial.

- *“I advise for mentally ill persons to try both religious and modern treatment options. For instance, there is Amanuel hospital, so it would be good if mentally ill people find treatment from there. Again families should take the mentally ill person to their religious center and let him/her get relief because people may be tensioned when they fail to go for worship or absent from their religious places. I think they feel free and be stable when they visit their religious place.” (a woman-IDI)*
- *“Orthodox Christians take to holy water and we have witnessed that many people got recovered from mental illness. Others who believe in Kalcha also do something related to that (worship Devil) and sometimes recover. Some go to hospital like to Amanuel Hospital…. People usually prefer the traditional and religious places; because; they believe in their faith and the second reason is, people don’t afford to take to hospital.” (a man-KII)*
- *“Around this community they prefer the religious service. But there isn’t a medical center so that is not a choice at all. Whether they like it or not they go to a religious solution and it is difficult to say they choose this or that.” (a man-IDI)*

The study participants were also asked from whom they may need care and what treatment options they prefer if they may face mental illness and most of them have reported that they primarily look for support from God and their family and the last option to be from health professionals.

- *“If I exposed for mental illness mainly God will help me, and also my family will be the possible care giver. I preferred to take spiritual means of treatment.´ (a woman-IDI)*

## Discussion

Community members’ perceptions toward mental illness and help-seeking behavior experiences were the major discussion categories of this research. Accordingly, although few participants have reported supportive perceptions, we have found gaps related to what mental illness means, how it results, what care and approaches a mentally ill person should get, what management and preference of treatment options are there, and experience of supporting mentally ill people. Through this study, all study participants have agreed that mental illness is a disease that leads to loss of consciousness, in which one person is unable to think properly as a healthy person does and fails to control himself and in which a person may misbehave, annoy the community and do unnecessary things. People assume that mental illness is very harmful and as a result mentally ill people are almost isolated from the community. Studies from Harar and Gimbi of Ethiopia have shown the same idea and other studies from Africa have also reported that people mismanage mentally ill people [8,10–15]. This is negatively influencing the health outcome to the affected people which highly demands strong work to shape the community’s mind for better change in the area.

People describe mentally ill people by using different names which are usually discriminatory terms like calling them mad (Ibid), unstable (tesh), unconscious (yenekele, aemrow yeteneka, nik) and others. Different literatures have also reported that using such terms indicate negative attitude towards the illness [3,21–23,25]. Hence, it is important to promote for proper term utilization in describing mental illness as per their respective categories.

Majorities of study participants have described that mental illness is a result of life complexities, stress, frustration, and substance use (chat, alcohol and others). This finding is supported by different study reports [6,7,25] and the similarity could be explained by comparable access of information related to mental illness. Effect of evil spirit resulted from sin and God’s punishment, hereditary and accidents or head injuries during birth were also part of the assumed causes for mental illness as reported by very few to few study participants. Similar conditions were reported from Kenya, Tanzania and other African places [3,8,9].

Mentally ill people may not be able to search how and where to get support and it is the family and the community who are supposed to approach them and find solutions to them. However, it is reported as it is usually missed and the responsibility falls on volunteer individuals once mentally ill people start to live in the street. As a result, many of mentally ill people are found to be neglected and their health conditions deteriorate from time to time rather than looking at promising things to better outcomes. Previous studies from Ethiopia [20,23,24] have also revealed such condition which demands serious attention to modify patients’ life.

Although people believe those mentally ill people should get all necessary medical and social support, what is found in the ground is that almost all mentally ill people are living on the street and feeding themselves by begging. There is no organized system that supports mentally ill people in the study area, as a result, they are usually dependent on very few generous people who provide them leftover food, coins, old clothes, and shelter (usually near to fences and verandas). As far as mentally ill people are not able to care for themselves, such exposure further aggravates their health problems because of poor diet and hygiene which could result in nutritional problems and again, the problem is not merely a lack of care or support to mentally ill people rather what makes it worse is there are conditions in which such people face abuse from the community members which aggravates their illness. It was reported that some people may laugh on them, insult or call them insane, approach them for fun and consider them as inhumane. Similar results were reported from Ethiopian and studies conducted from Indonesia and Cambodia [10,16,17,20,23–25,32].

In this study, most have suggested to take mentally ill people to their respective religious places and get prayer and other support. Similar results were reported from Gimbi, Jima and Silte of Ethiopian places [10,23,25].The reason why most have preferred religious places is because they believe the causes of mental illnesses are more of spiritual related and the solution is also from God. Again, lack of healthcare facility that provides medical services to mentally ill people was also mentioned to be the reason to prefer religious places. Some others have revealed that taking mentally ill people to both religious places and healthcare facilities will be beneficial.

### Strengths and limitations of the study

This study involved a number of study participants with diversified backgrounds who could well explain the issue of interest and the research team was also composed of different professions including public health, psychiatry, psychology, and social work which gives strength to this work. However, this study has limitations in terms of failing to directly approach mentally ill people or those who get recovered from it to get their real experiences.

## Conclusion

This study revealed that the way people perceive and describe mentally ill people, the way they approach and the help-seeking behavior was discriminatory. Although some family members try to take the mentally ill people to respective religious places and rarely to health facilities in the initial phase of the illness, later almost all of mentally ill people are usually left to live in the street and face different discriminatory reactions and isolation from the community member. The study has also found that there is no organized system for how mentally ill people get support, care, and treatment in the area rather their fate was found to be determined by few number of people who are willing to provide them food, clothes, and shelter.

### Recommendation

Based on the study findings; we shall recommend to the respective government health bureaus to create a system, especially to make mental health services accessible to the community and design strategies to foster communities’ awareness regarding mental illness. Again it is important to work with community leaders, religious leaders, and other influential persons to change communities’ perception. We also recommend other health agents to create an organized system that provides mental health services in the area.

## Supporting information

S1 File Transcribed data sample. (ZIP)

## Abbreviation:

IDI: In-depth Interview
KII: Key Informant Interview.
