# Supplementary material for: “People are shortening the lifetime of mentally ill persons”; Community’s perception towards mental illness and help-seeking behavior in Bench Sheko, Sheka, Kaffa and West Omo zones, South West Ethiopia, 2021
Source: PLoS One. 2025 Apr 29;20(4):e0320740. doi: 10.1371/journal.pone.0320740 (PMC12040187; doi:10.1371/journal.pone.0320740)
Supplement: S1 File — (ZIP) [file pone.0320740.s001.zip › Transcribed data sample/Interview data (A).docx]

**Research title: *Community Perception and Help-seeking Behavior Towards Mental Illness and Its Associated Factors among Bench-Sheko, Kaffa, West Omo and Sheka Zone***

Region: South West Ethiopia regional state

Interview category: In depth interview

Setting: Rural

Key:-

I:-Interviewer

P:-Participant

I: First thank you for your willingness to participate in the interview. Start by introducing yourself.

P: I am a youth who works in a government office, I am serving the public and the government, I am educated and graduated. I am …… years old. I have a degree in water engineering and I am a water engineer in the zone’s water department.

I: What about your family situation?

P: I live far from my family, I live alone.

I: As I told you before our discussion is about thoughts regarding mental illness, support, causes and influencing factors. What is mental illness?

P: I think mental illness is a bigger illness compared to other diseases because the motor part of a human being everything is on the mind. If that part is damaged, recovery to normal may take time or it is not like other disease you get treatment and solve the problem. Once you deviate from the normal returning to that requires a lot. So I think mental illness is a big and scary illness.

I: Where did you hear the information you told me?

P: I read books and hear radio and worked on mini-media and I heard from these sources and you hear the community talk about people with mental illness and how it started. That is how I learned about the illness.

I: What are the signs of mental illness?

P: I didn’t study about this but from my view, you will experience behavior that is different from a normal human being, you will see them when they happen for example:- normal people are expected to dress, eat and talk in a certain way and you observe things that are different from that with mentally ill person. They are not normal when they sit, talk and walk.

I: Do you have a family member with mental illness?

P: No.

I: How does the community describe mental illness?

P: Their description is kind of intensifies the illness. People may experience mental illness due to stress but people describe it via sarcasm that will increase their illness. They don’t see it positively. They stigmatize, tell people to distance themselves because he is insane. They belittle and pressure his illness.

I: What do people in your locality call people with mental illness?

P: In Local Kefigna they call it *Gecho,* it is like calling insane. They use sarcasm every time goes around or talked about that person. They insult them and call them insane.

I: How close is the community to people with mental illness?

P: all I see is people trying to intensify their illness rather than helping to improve their situation. It is hard to find who are ready to serve other people at this time. There is a high chance the people with mental illness will recover to normal if people gathered them and cared for them and given them love, but we don’t see that. Rather we see people making them angry and adding illness to their disease and shortening their life time. I don’t think people who have the knowledge and gift to treat those people and save them are born yet.

I: Do people help the mentally ill in your locality?

P: I don’t think they do, I think their family is the only once worried about them. Sometimes loyal friends and relatives may help on taking them to a holy water or to a medical center. I don’t think others participate like that and I don’t see it.

I: How does the community think mental illness start?

P: When you are born in a community that is traditional and uneducated being a different person is a problem. In our community for example smart students loose themselves when they hear that somebody has done some magic on them. even business people who have a gift for it and work hard to improve themselves and their country are not liked by people and they take revenge by making lose their mind. People who hurt others like this are those who have a narrow and small mind. Because the community don’t like people who are created different and are active in education or trade. They go to a witch and do something to make that person loose his mind that is what I observe in our locality. I do think you will be hurt if you are a different person.

I: How does the community view a person with mental illness?

P: There isn’t a normal form of expression. In the locality other than remembering what he was yesterday, they belittle, torture, and tell you will not be normal repetitively. There is no one who will think about what you did in the past and try to return the favor now. I don’t think an institution that does do something and people do not have the understanding to do that. If people understood that I think they will have a positive thing for people with mental illness. But given that awareness is not done, the community is yet to say we should help him and treats him and he will return to normal.

I: What do you think must be done to solve the above problems?

P: I do think there are institutions in big towns that provide treatment for mentally ill people; I do think there are a few as a country. But when you come to Keffa, it might not even come in the next forty or fifty years. If there were institutions they will teach the community, since there isn’t an institution those people who are close to the media have an understanding that it can be treated. Those at the kebele level do not understand. So as a government this institutions should go to the local level. They may create awareness and the religious institutions can work with the government since most of the mentally ill can be found in religious centers. The religious institutions can build a center that treats the mentally ill other than holy water and that will improve the situation.

I: How do people view mental health institutions and are there any around here?

P: They might go to the hospital or to a clinic and there is a lot of medication for the mentally ill and there are psychological treatments but in our locality they go to a holy water for treatment. Some who have the capacity may go to Addis Ababa. But in our locality I don’t get you will get a medication even if you go to a hospital or clinic because there are a lot of steps he has lost his normal mind and if it is related with witch craft then he will be cured in holy water depending on his faith. Other than that there is nothing we see in relation to institutions.

I: Which one does the community choose between religious institutions and medical institutions and why?

P: I guess it depends on their economic level and their faith. If they are faithful and give everything to god they will go to a holy water site. If they have economic capacity they will go to a health setting and religious institution and holy water will be a second option. In our locality you wouldn’t say they have good economic situation and you can’t say they have similar faith and there are those who live their entire life without seeking any treatment and who spend their day chained at home. Depending on their belief and their consciousness about the issue, even relatives may take to a holy water but taking them to a medical center this hasn’t grown yet. Most of them are those who are tied at home hidden and ashamed that the community sees hem negatively. If they believe they take them to a holy water and if they have money they take them to a health center and these are the three options.

I: Explain care for the mentally ill person with example.

P: there is care, we see in our town Bonga people return to normal with small treatment. After they become normal the treatment doesn’t continue and they regress to their previous situation. If there was work in relation to institutions and the government there are people who would return to normal with little effort. If they are not normal you take them to some place, you cloth them, and follow its own step, we might not get change in a week or month, but if there were people who will tolerate the challenges and continue to help them then they will see results.

I: How and by whom can the caregiving you told me provided?

P: By volunteers and youth who work on this issue and people who decided to contribute on this issue. Even if the government builds a center there still will be a need for someone to wash and care for this people. We will need people in those centers because it is full of challenges that you need to consider and decide to treat people equally and wash and groom them. You don’t do this by obligation rather you do it for your mind and volunteering and ready to serve others. Doing good is for yourselves and you would get it from God and he will give you riches and peace. You can only serve if you get in to it with this decision. Even if the government demand to do this I don’t think they will do equal job with those who have volunteered to serve.

I: Have you given care and support?

P: I don’t have bad intentions towards this people. I do give what I have to them, you meet them in the road or around your home and what do they need, they need money and I have supported them in that even though it wasn’t much. But I don’t have done anything other than that because I dint decide to do things, but when they come by chance while I am eating lunch, and they come and say they are hungry and thirsty I support them.

I: Have you thought you might get mental illness?

P: I don’t know. I do think that our mind is human beings biggest asset and if we lose that I don’t think we can live. I don’t think I will do things that will take me to stress and mental illness because I work all the time, reading books sooths me. I do think people should live with limit; I am limited on the identity I was built in. I don’t just think that I should get something without working for it, when it fails I start to worry and worry is like an ocean it widens itself and it is difficult to get out of it. If you are in a worry you will lose yourself. Why? I want to be happy today and live while treating myself. I don’t think I have a way towards that if I move with caution unless I make a mistake. God protect us.

I: Amen. Like you said if it happens in accident who do you think will help you?

P: first God and my family and then my close friends will help me. Though they couldn’t be like God and my family. In this world, I think I will pressure my family first, then God will visit me and then my friends will help me if they remember me.

I: Like you from whom do you think the community prefer to get this support?

P: There is nothing that will come before family, there is nothing. Even close people and relatives can’t be like parents and siblings, they will get bored. It is a boring thing and people get depressed. I don’t think people who are not related to me in blood can give that whole time that may take many years and take care of me. If you are lucky and have mother and father or relatives otherwise you will be thrown outside like the people we see now. I do think it will be like that and it is a scary thing.

I: Ok. What do you think must be done about mental illness given the current situation, by the government?

P: The government should include issues of mental development like we say washing hear and cloth, our mind requires care and it grows with age and mind must be treated and these issues must be included in education starting from kindergarten. If people know that the mind is a motor that grows as we grow and become aware of the causes of mental illness, if they live with limits and if they fear God and respect the law they don’t have a reason to become mentally ill. Religious or spiritual institutions which teach life after death, I think can only go to heaven if they were healthy in earth because if they lose their mind and start to behave inhuman I don’t think these have a chance for redemption. When you are a believer there are things you do like fasting and praying, tithe etc and these people do not do this. The church should worry because to keep her children mentally well and as she teaches morality and ethics, it should also cover mental health and physical health. I do think depending on the religious institution.

I: If there is an idea you think we didn’t cover you should raise it.

P: In bigger cities we see a lot of things, people recovering and working and enjoying their life and serving the community. There are people who sacrifice themselves for the service of others and how do they come to this situation? Who are this people who treat the mentally ill? In the locality this things are not known and creating awareness will be good. I think education at kindergarten should cover what this people do because it will be engraved on them. The mind is the main motor of a human and I do think the government and the country should work on keeping it healthy because it would have multiple benefits. If there are not normal people the country will not be a country; normal people are the once who go to war to defend a country. Even though we can’t create the good people in those cities here but the sheer awareness that it can be done like that is better. Now there might not be change but the people who will grow up listening about this will become service providers.

I: Ok. I have finished my questions. The main issues we covered are many and good. Thank you for your participation and cooperation.

P: Thank you. These are the things I heard and if I have read about mental illness I would have been more helpful. Thank you.

I: Ok. Thank you.
