# Supplementary material for: “People are shortening the lifetime of mentally ill persons”; Community’s perception towards mental illness and help-seeking behavior in Bench Sheko, Sheka, Kaffa and West Omo zones, South West Ethiopia, 2021
Source: PLoS One. 2025 Apr 29;20(4):e0320740. doi: 10.1371/journal.pone.0320740 (PMC12040187; doi:10.1371/journal.pone.0320740)
Supplement: S1 File — (ZIP) [file pone.0320740.s001.zip › Transcribed data sample/Interview data (B).docx]

**Research title: *Community Perception and Help-seeking Behavior Towards Mental Illness and Its Associated Factors among Bench-Sheko, Kaffa, West Omo and Sheka Zone***

**Region: SNNPR**

**Interview category**: In depth interview

**Interview ID:**

**Setting: Rural**

**Key:-**

**I:-Interviewer**

**P:-Participant**

I: Ok thank you! Please introduce yourself? your age, education, marital status

P: Okay, thank you!

I: Like I told you earlier, this interview will focus on perception on mental illness, help seeking behavior and factors contributing to mental illness. So, from your experience, how do you define mental illness?

P: personally, I think mental illness can happen through different factors including life stress, unable to achieve once goal an d other life obstacles coming together in a person and push him/her in to experiencing mental illness.

I: Where do you get all these information about mental illness?

P: From reading, watching movies and by directly observing people with mental illness in my surroundings.

I: From your understanding, what are the symptoms of mental illness?

P: There are different kinds of mental illness. Some of them are agressives who chase people around them. Some of them are very quiet who mostly detach themselves from the community and who have less apetite for food.

I: Do you have a family member with mental illness?

P: No, I don’t have mentally ill family member.

I: How do the community percieve and define mental illness?

P: The community don’t have a good attitude for mentally ill people. They are not ready to accept and support them. They are always running away from mentally ill people.

I: How do the community call mentally ill people?

P: Usually thay are called as *ebd, chelel* etc

I: How do you explain the communitys closeness to mentally ill people?

P: As much as I know , the community is not ready to approach mentally ill people. I never seen or herad of a person gettin close and provide care and support for mentally ill person.

I: What kind of care and support are provided by the community?

P: During holidays , they are provided with food and some people repairs thier house too.

I: Have you ever provided care and support for people with mental illness?

P: To be honest, I never provided care or support for people with mental illness.

**0:05:00**

I: From your understanig how do the community percieve the factors contributing for mental illness?

P: too much stress, hereditary factors, accidents like car crash. All these might contribute for mental illness.

I: How do the community percieve people with mental illness?

P: The community have stigma towards people with mental illness. They are not interested to seek out what they wanted and to provide support for people with mental illness.

I: What do you think should be done to make the situation favorable for people with mental illness?

P: getting closer those with mental illness is the first. Then we can undeesrand thier past life , what contributed to thier situation. Based on that we can easily understand thier nedd provide them sith care and support.

I: How do the community see mental health treatmet in this locality?

P: As much as I know, there is no mental health centers in this area. Even in ethiopia, I only know amanuel mental health hospital.

I: How do the community percieve treatment alternatives?( religious, traditional, mental health service)

P: Mostly the community prefers to attend religious programms to get treated. If they are orthodox christians, they will go to holly water. Other religious followers also attend thier respective religious activities to get treated from mental illness. The community do not think that modern mental health service can treat those with different mental health problems.

I: What should be ways of care and treatment for people with mental illness?

P: First, its important to identify thier needs. Starting from cloth and food , we can then focus on providing them with treatment , either religious or service from mental health centers.

I: Who should provide all these care and support for people with mental illness?

P: The community can provide care and support. Health professional in different health settings can also provide care for people with mental illness.

I: have you ever provide care and support from mentally ill person?

P: No, I didn’t.

I: Have you ever thought of personally experience mental illness in the future? Who do you think will provide care and support, if you experience mental illness?

P: This is a difficult question. We can not be sure about what will happen tomorrow. So, its not possible to say wheteher I will have mental illness or not. Its in the hands of God. If by chance I experience mental illness, I think it will be my family who will provide care and support for me.

**0:10:00**

I: From who do the community prefer to get care and support in case of mental illness?

P: I think , they would prfere to get care and support from thier friends , relatives and family members.

I: Regarding mental illness, by considering the reality on the ground , what should be done to make the situation better? (community, govt)

P: The govt. Have a lot of assignments to do. Its expected from the govt. to construct houses for people with mental illness. Usually, this kinds of intiatives are performed by individuals like the man who established Mekedonia. This person should be a role model for able bodied individuals and for the government to expand mental health related service facilities.

I: I have finished my questions. But if you have anything to add on this issue, you are welcome.

P: I think I have said what I am supposed to say. But, we should give emphasis for the problem. We should approach and provide care and support for people with mental illness.

Starting from today, I will try my best to get close those with mental illness and provide them support to may capabilities.

I: I have finished, Thank you for your participation!

P: Ok! Thank you!
