# Supplementary material for: “People are shortening the lifetime of mentally ill persons”; Community’s perception towards mental illness and help-seeking behavior in Bench Sheko, Sheka, Kaffa and West Omo zones, South West Ethiopia, 2021
Source: PLoS One. 2025 Apr 29;20(4):e0320740. doi: 10.1371/journal.pone.0320740 (PMC12040187; doi:10.1371/journal.pone.0320740)
Supplement: S1 File — (ZIP) [file pone.0320740.s001.zip › Transcribed data sample/Interview data (C).docx]

**Research title: *Community Perception and Help-seeking Behavior Towards Mental Illness and Its Associated Factors among Bench-Sheko, Kaffa, West Omo and Sheka Zone***

Region: South West Ethiopia regional state

Interview category: In depth interview

Interview ID:

Setting: Rural

Key:-

I:-Interviewer

P:-Participant.

I: Ok! Thank you! As I said earlier our discussion is about mental illness thought, support seeking, and causing factors. Explain these issues.

P: Ok! In our locality they attach it to belief or many traditional things, which is what is accustomed. But what I think is mental illness is when someone is different from what a healthy human being does including acts, speech. Other than that they show different symptoms.

I: What for example?

P: For example if he was walking with cloth yesterday, today he will start by taking of his cloth. He will start chasing people with stone. He will reach to biting any one he encounters, or catches. These are the symptoms.

I: Where did you hear this?

P: I didn’t hear it; I saw it in our locality. They are segregated from the community and I see them in the streets when they insult, scream, hit and chase people.

I: What kind of signs are there for mental illness?

P: Mental illness doesn’t vary by locality, mostly it is similar. When mental illness starts the person will change from his previous situation. The changes are taking his cloth of, he can’t communicate, you can’t talk to him like you do in the past, he throws stones, there is also biting, insulting , screaming. These signs identify them from the community.

I: Ok! Was there any family member with this illness? If there is, tell me your experience.

P: No. there isn’t.

I: How does the community describe mental illness?

P: In similar way. As I said earlier in relation with different beliefs, they say it is because of the calling of the Ark and somebody did something to him. They believe in these things. They don’t connect it with medicine. Most of the time, they connect it with belief.

I: What do they call a person with mental illness?

P: He is called mad (she said *Ebid*).

I: How is their connection and support?

P: There isn’t. But rarely some volunteers take them to a holy water sites, to different healing places. This is rare, if everybody did this, there will not be many people with mental illness. There are few people who take them to holy water sites to be baptized.

I: Based on these facts, you are saying it is not common?

P: Yes.

I: Why? Do the people distance themselves? Do they fear them? What makes them distance themselves or misunderstand.

P: First, the people distance themselves because they think they will hit and bite them. In addition they worry the illness will transfer to them if they bite them. There is lack of awareness in the community so people who work on this issue should move around the locality teach the community about it. Unless this is done the communities interest to get close and understand is small.

I: What does the community believe causes mental illness? From family? Or from pathogenic microorganisms? Or from Cult (Ba’ed Ameliko)? Do you understand me? I mean what does the community think cause the illness?

P: In our locality they say it comes from belief in cults and they also believe it is transferred through heredity.

I: The community thinks this much?

P: Yes.

I: When I asked you about what the community thought causes mental illness you told me that from heredity, what about pathogenic microorganisms?

06:00

P: No, it doesn’t transfer. There is no one who thinks this much.

I: How does the community view a person with mental illness?

P: The community secludes and distances. Even after he is cured their reaction will push him to the illness, they don’t trust him. The seclusion is very common in our locality. When someone is getting better and comes to the community, the people have difficulty trusting him.

I: What should be done?

P: Concerned bodies should work together. Psychiatrists and health bureau in connection with, government offices, labor and social affairs should come dawn and together orient the community. We should do awareness creation in the form of training or in poem or other forms and media campaign. So, the community will not seclude.

I: Ok! Thank you!

I: how does the community see health services for the mentally ill?

P: Small portion of the educated think that medication is better, only 30 percent of them think like this. The rest believes that the solution is Holy water and other different belief institutions, other than that he can’t be cured.

I: Is there health institution?

P: There isn’t in our locality.

I: You said the is no health facility (talking about mental health facility)

P: Yes. There isn’t a rehabilitation center around us so that we might put those in our surrounding. Those with better economic power can take to Amanuel in Addis Ababa and get cured. If there were rehabilitation centers and if those centers have trained professionals, there will be a chance for early treatment and recovery. Because, when the mentally ill join the street the community does a lot of things they don’t like and make their problem worse. There has to be a rehabilitation center in every locality.

I: Ok! Which one do people prefer from traditional treatment and modern medication?

P: In choice our people think he will recover. They will try both treatments. But after we provide awareness in the community they will prefer medication.

I: In current situation which one do they prefer?

P: They take them to their respective belief institutions and other cult.

I: Why is that? Is it due to lack of awareness?

P: There is huge lack of awareness.

I: Ok! Thank you! Can you tell me about care for a person with mental illness with example? What is needed for a person with mental illness?

P: Step by step getting close to them, while we are close not doing the things they don’t like. Some people with mental illness do not like red things, there are such things. All people with mental illness have things they like and they don’t like, so we should avoid the things they don’t like, give them food, cloth them, wash them, if we get close like that they will not be that much difficult, they will come to a better way.

I: How and by whom is the caregiving provided?

P: Mostly it is by family, when it is above the capacity of the family the community should have done this. But that is not being done and this gap came from lack of awareness, this will be done after the community acquires awareness.

I: Have you provided care and support?

P: Yes. By our bureau in relation to Covid we took them to a rehabilitation center by force and treated them but then came procrastinating and we released them.

I: Ok! Thank you! Have you thought you may experience mental illness?

P: Yes.

I: How? If it does who do you think will help you?

P: We can’t say that mental illness will not happen to me, we might experience it. Even if it is for one second, at times of huge stress and issues there are things we create, we even reach to a point where we forget where we are. When these kinds of events increase their time, it turns to mental illness. We are healthy and walking that doesn’t matter rather when we talked about it in relation with cults but mental illness also comes from not relaxing and worrying all the time. So it is difficult to think it will not happen to me.

I: If it happens who would help you? Who do you prefer to support you?

P: By that time I will not be able to choose. I think my family will help me specifically my husband. But if I start recovering I think I will choose to go to a health facility and get treatment appropriately. Going to a health facility is also giving rest for the family because the health professionals will be taking care of me.

I: From whom does the community think to get support?

P: The community is happy to get the support from anyone because they want to be healthy. But there isn’t such support so they seek those who are close and support them. But there isn’t many people providing support. Only few volunteers do this.

I: Ok! Thank you! What should be done about mental illness by the government, by individuals and by other civil associations?

P: As I put it earlier, this is not going to be left for those who have concern on the issue or those called stakeholders. It can’t be assigned for the government or individuals as you put it, it doesn’t have stakeholders because everyone is a stakeholder. But as people struggle to improve their livelihood they don’t focus on such issues, so there are sectors or agencies that should work with focus on this issue. There are things that should be done starting from the health ministry including rehabilitation centers, medication, assigning professionals and raising awareness in the local level.

I: ok. What is expected from individuals?

P: ok. At individual level, one person can do a lot of things. If he can befriend the person with mental illness that is good thing, if he can’t do that, if they live in one facility that person can support in fulfilling their food and clothing needs. He can also cloth and wash those who are in the streets.

I: What is expected of religious institutions? Explain it for me.

P: If they have any medication, religious institutions should tell them it is ok to take it. Because when people go to a holy water site they want to know who cured them for that purpose they leave their medication. The religious institutions should make sure that people take their medication while they are getting the baptism treatment.

I: I have finished my questions. Is there any additional issue you think must be raised?

P: the additional thing I have is that gathering data like this shouldn’t be the end of it. It should be implemented. There should be a treatment center in this locality for the mentally ill. At this time, those who can recover in a short time are spending their whole life with mental illness. So recovery centers must be built in our surrounding.

I: Ok. I have finished my questions. The main ideas you raised are mental illness around the community is called mad (he said *Ebid*) and treated as such, when he walks naked and does other things the people treat him based on that. You said people fear them because they hit them with stone and have different thought, and the people do not support them and seclude them. You said that the illness comes from heredity and believe in cults. You also said that people distance themselves seclude them. You said that people should befriend people with mental illness and stop secluding them and the family should provide support because they are close to him. You added that there is misunderstanding among the community. You commented that we shouldn’t stop on gathering data rather we should put it in to practice. This is research and we will communicate the information to concerned bodies. Thank you very much for your participation.

P: Ok! Thank you!
