# Supplementary material for: “People are shortening the lifetime of mentally ill persons”; Community’s perception towards mental illness and help-seeking behavior in Bench Sheko, Sheka, Kaffa and West Omo zones, South West Ethiopia, 2021
Source: PLoS One. 2025 Apr 29;20(4):e0320740. doi: 10.1371/journal.pone.0320740 (PMC12040187; doi:10.1371/journal.pone.0320740)
Supplement: S1 File — (ZIP) [file pone.0320740.s001.zip › Transcribed data sample/Interview data (D).docx]

**Research title: *Community Perception and help Help-seeking Behavior towards mental illness and Its Associated Factors among Bench-Sheko, Kaffa, West Omo and Sheka Zone***

**Region: SNNPR**

**District: Bachuma**

**Interview category**: KII

**Interview ID:** KII-Social1

**Setting:** Rural

**Key:-**

**I:-Interviewer**

**P:-Participant**

I: Ok thank you! please tell me your age, about your education level and occupation. **(Participant is male)**

P: Ok thank you!

I: Ok as I told you earlier; our discussion point is about Community perception and help seeking behavior towards mental illness and its associated factors, so please tell me what mental illness means.

P: Mental illness is a disease in which one person is unable to think properly as healthy person and fails to control himself.

I: What symptoms do these mentally ill people do have?

P: A mentally ill person may misbehave, annoy the community and do unnecessary things. So if someone does odd things which are uncommon and an acceptable by the community; the person would be considered as mentally ill.

I: Ok was there any one who has experienced mental illness among your family?

P: No, there is no one.

I: How do the community members see mental illness?

P: People usually call a mentally ill person as Mad (Abdual or rasun situal, ye aemro ekil).

I: How does the community approach and support the mentally ill people?

P: We are not seeing support to mentally ill people from the community. Usually attention is given to support poor people(economically lower class people) than mentally ill people both from the government and the community. The community don’t approach mentally ill people because of fearing attack or else.

I: What are causes of mental illness as per the community’s thought?

P: It occurs incidentally and some referred the cause to be evil spirit related issue like likift and muart (sew be lela sew lay yehone neger/metet maserat). Again some relates it with heredity.

I: What should be done for a mentally ill person? How the communities think?

P: There is no effort to support mentally ill people rather most refuses to approach them.

I: Where do people take a mentally ill person for treatment?

P: It depends with religion of the family; some may take to holy water and others to church

I: What health facility is there which may support mentally people?

P: There is no health facility around us but we have information about health facilities which may give medical services for mentally ill people.

I: Where people prefer to take a mentally ill person for treatment?

P: People usually prefer the traditional and religious places; because since most people relate the cause with evil spirit, they also think that the solution would be from religious or traditional places (sew metet kaderegebet betsebel yichohal/yawotal).

I: What care does a mentally ill person need? Please tell me examples of cares.

P: Multiple care including food, cloth and shelter are needed but our community fears mentally ill people and usually refuses to approach them.

I: Have you ever given care to a mentally ill person?

P: I try to approach them. For instance; our community doesn’t approach epileptic and mentally ill people due to fear of transmission while actually the case is not communicable, but I approach them and let them to participate in works they are able to do like fetching water.

I: Do you think as you may face mental illness?

P: Since I do things with plan and take care of myself, I don’t think I face mental illness.

I: From whom do the community prefer to get a support or treatment; Modern or traditional or spiritual?

P: Usually they prefer from religious and traditional areas.

I: What should be done regarding mental illness from government, NGOs and other stakeholders?

P: We have to create awareness among the community members in order to foster culture of care. Again would be good if we establish centers like Mekodenia to accommodate mentally ill people and facilitate for care and treatment. All should work to manage the case and aware the community.

I: Do you have any additional points?

P: I believe such researches could be solution indicators and I look forward for that.

I: I have finished, Thank you!

P: Ok! Thank you!
