# Supplementary material for: “People are shortening the lifetime of mentally ill persons”; Community’s perception towards mental illness and help-seeking behavior in Bench Sheko, Sheka, Kaffa and West Omo zones, South West Ethiopia, 2021
Source: PLoS One. 2025 Apr 29;20(4):e0320740. doi: 10.1371/journal.pone.0320740 (PMC12040187; doi:10.1371/journal.pone.0320740)
Supplement: S1 File — (ZIP) [file pone.0320740.s001.zip › Transcribed data sample/Interview data (E).docx]

**Research title: *Community Perception and help Help-seeking Behavior towards mental illness and Its Associated Factors among Bench-Sheko, Kaffa, West Omo and Sheka Zone***

**Region: SNNPR**

**Interview category**: KII

**Setting:** Rural

**Key:-**

**I:-Interviewer**

**P:-Participant**

I: ok as I told you earlier; our discussion point is about Community perception and help seeking behavior towards mental illness and its associated factors, so please tell me what mental illness means.

P: Mental illness is a disease which made a person to be odd from normal persons and engaging in unnecessary conditions. So such people are known to be as mentally ill persons.

I: What symptoms do these mentally ill people do have?

P: The first thing is loss of consciousness and deviating from the community’s norm like living in the street, walking bare body and not acting as healthy person.

I: Ok was there any one who has experienced mental illness among your family?

P: No, there is no one.

I: How do the community members see mental illness? How do they describe mental illness and how they call a mentally ill person?

P: Usually mentally ill people are known as mad or the community call them saying mad (Ibid)

I: Do mentally ill people get support or care from the community?

P: Not common but sometimes people try to tie and take for holy water or health care service but most live on the street without getting care and even facing stigma.

I: What are causes of mental illness as per the community’s thought?

P: Mostly mental illness is seen as God’s punishment (Ye Egziabher kuta or mergem). So usually mental illness is associated with evil spirit resulted from sin and God’s punishment.

I: What is community’s perception toward mental illness and mentally ill persons?

R: It is stigmatizing and there is no good approach and support which may help them to get treatment.

I: What should be done for a mentally ill person? How the community thinks?

P: As we hear about; it has treatment and could get recovery but on the other hand, the treatment center is not accessible and cost is not affordable. Again people lack awareness about its treatment and where to get it.

I: Where do people take a mentally ill person for treatment?

P: Mostly people prefer to go for holy water and Protestants do also take to churches for praying. Otherwise it is not common to take mentally ill people to health facilities.

I: Why do you think people prefer the religious places?

P: The first thing is; the norm or the trend they are familiar with and the second thing is; people don’t have awareness and information about the health care services related to mental illness.

I: What care does a mentally ill person need? Please tell me examples of cares.

P: Usually people who have strong family get support otherwise most live in the street.

I: Have you ever given care to a mentally ill person?

P: I try to support them in providing cloth, food and even contribute money and send them for health care service.

I: Do you think as you may face mental illness?

P: Why not because I am also a human being, so it my happen incidentally.

I: Whom do you think may help you if you face mental illness?

P: My family will help me.

I: From whom do prefer to get a support or treatment; Modern or traditional or spiritual?

P: Firstly I prefer the religious support.

I: What should be done regarding mental illness from government, NGOs and other stakeholders?

P: Everyone should play its role. The government, NGOs, and the community members should contribute their parts. Awareness creation should be there among the community member and every should know as mental illness could occur among anyone and could also be treated if get adequate treatment.

I: Do you have any additional points?

P: No.

I: I have finished, Thank you!

P: Ok! Thank you!
