# Supplementary material for: “People are shortening the lifetime of mentally ill persons”; Community’s perception towards mental illness and help-seeking behavior in Bench Sheko, Sheka, Kaffa and West Omo zones, South West Ethiopia, 2021
Source: PLoS One. 2025 Apr 29;20(4):e0320740. doi: 10.1371/journal.pone.0320740 (PMC12040187; doi:10.1371/journal.pone.0320740)
Supplement: S1 File — (ZIP) [file pone.0320740.s001.zip › Transcribed data sample/Interview data (F).docx]

**Research title: *Community Perception and Help-seeking Behavior Towards Mental Illness and Its Associated Factors among Bench-Sheko, Kaffa, West Omo and Sheka Zone***

**Region: SNNPR**

**Interview category**: In depth interview

**Setting:** Rural

**Key:-**

**I:-Interviewer**

**P:-Participant**

I: This interview is about perception on mental illness, help seeking behavior and contributing factors. So, how do you define mental illness?

P: mental illness can be defined in different ways. Those with mental illness, act differently than the community or if they are unable to think and act irrationally, we call them mentally ill people. But this should be confirmed by professionals.

I: How do you get all these information about mental illness?

P: I read different books and i get mental illness related infromation from those books.

I: From your experience , what are the symptoms of mental illness?

P: Being unable to talk properly, being nakedacting against the valuse of the community. There might also be other symptoms as well.

I: Do you have a family member with mental illness?

P: No, I don’t have one!

I: How do the community percievce and explain mental illness?

P: in fact ,in our community perception on mental illness is a bit different. Sometimes if you don’t agree on some ideas with others, they might consider you as a mentally ill person.That is totally wrong. The community call a person mentally ill if they act against the valuses and norms of the society. But , I think it should be confirmed by the mental health professionals to make sure whether someone is mentally ill or not.

I: How do the community call those people with mental illness, its relation and support?

P: Usually they call them as *ebd*. The community , most of the time, do not apprach metally ill people and thier associeted stigma with it. But I think its better to understand thier situation and provide the appropriate support.

I: How do the community percieve causes to mental illness? (drom family , disease transmitting insects, worshipping the devil)

P: Social science researchers said that mental illness might be caused by economic challenges, loss of a father, mother a child or loved once and other unexpected events contributes for mentall illness.

**00:05:00**

I: How do the community percieve people with mental illness?

P: In Our community its very common to stigmatize people with mental illness. Even families of mentally ill person hides that individual since the community relates it with a curse or evil thing.

I: What should be done to avert this perception from the community?

P: I think the community should undesrtand the problem of mentally ill people and support them. They can support them financially, emotionally by showing empathy.

I: How do the coomunity percive treatment for mental illness?

P: Mostly people with mentall illness goes to religious places like holly water. But its better first to talk to mental health professionals and after that its possible to go to holly water.

I: Is thier any mental health centers available in this area?

P: There is no mental health center in here. There is only one mental health hospital in ethiopia, Amanuel. People mostly goes to witches to address mental illness issues.

I: How does the community percieves treatment alternatives?

P: Those who are protestants will attend prayers and those who are orthodox will go to the holly water.

I: Why do you think people prefer this alteranatives?

P: This is becouse thier is no mental health centers in this area. In addition, thier is also a misconception regarding mental illness and becouse of that people prefer to attend religious events and witches than going in to mental health centers.

I: How do you describe care and support for people with mental illness? What are needed for PWMI?

P: First, its important to get close in to people with mental illness and ask them thie need. Linking them with mental health professionals is also important. The community should also avoid a sitgmatized attitude towards people with mental illness.

I: By whom are those care and support provided for people with mental illness?

P: Mostly by family members, religious leaders. Sometimes, mental health professionals also might give care and support for people with mental illness.

**0:10:00**

I: Have you ever provided care and support for people with mental illness?

P: Sometimes I just listen to what they say becouse I believe being heard is avery importants thing for them.

I: Have you ever imagined that you might experience mental illness in the future? Who dou you think will provide you care and suport?

P: Mostly I read psychology books and before thigs get out of control I will manage them effectively. Becouse of this I don’t think I will have mental illness in the future. But if by chance I get the problem, I think mental health professionals will provide care and support for me.

I: Considering the reality on the ground, what should be done regardinf mental illness by the govt., individuals, community?

P: The govt. Should expand mental health centers, distribute mental health specialists. The community should also raise funds to organize mental health centers. The community also should avoid its stigmatized attitude towrds people with mental illness.

I: Do you have anything to add regarding mentall illness and related issues in your environment?

P: In our country, many people have mental health problem. I think its better to get close to people with mental illness and provide care and support as much as we can.

**0:15:00**

I: I have finished my questions. Thank you very much for your insightful explanation about mental illness. They are really useful!

P: Thank you for giving me the chance to express my view regarding mental illness.

Thank you!
