# Supplementary material for: “People are shortening the lifetime of mentally ill persons”; Community’s perception towards mental illness and help-seeking behavior in Bench Sheko, Sheka, Kaffa and West Omo zones, South West Ethiopia, 2021
Source: PLoS One. 2025 Apr 29;20(4):e0320740. doi: 10.1371/journal.pone.0320740 (PMC12040187; doi:10.1371/journal.pone.0320740)
Supplement: S1 File — (ZIP) [file pone.0320740.s001.zip › Transcribed data sample/Interview data (G).docx]

**Research title: *Community Perception and Help-seeking Behavior Towards Mental Illness and Its Associated Factors among Bench-Sheko, Kaffa, West Omo and Sheka Zone***

Region: South West Ethiopia regional state

Interview category: In depth interview

Interview ID:

Setting: Rural

Key:-

I:-Interviewer

P:-Participant

I: As I told you earlier our discussion is about thoughts regarding mental illness, support, causes and influencing factors. What is mental illness?

P: Mental illness is being mentally abnormal or having a mind that behaves different from normal person.

I: Where did you hear this information?

P: From health institutions, media and the community I grow up in and by observing people with mental illness.

I: What are the signs of mental illness?

P: There are different types of mental illness, there are those who walk naked, those who talk alone, those who hurt people, those who scream, these are some of them but there is more.

I: Do you have family member who was mentally ill?

P: No.

I: What about in your surrounding?

P: There are a lot. I have seen many and there are currently.

I: How does the community explain mental illness? Explain.

P: When someone does something that is different from a normal person and from his action and situation different than a normal person some may think he is crazy some others may see him in pity. There are also people who understand begging from them how it starts, because of reading they know. Each one explains mental illness differently depending on its own understanding.

I: What do community members call people with mental illness?

P: They may be called mentally ill or crazy, *Qe’wes*. They also say in general as those ill (*Beshitegna*).

I: How close are they?

P: It depends on the people there are who are close there is who flee at the sight of them.

I: Why do they flee when they see them?

P: Depending on the type of illness there are mentally ill people who hurt others, so they flee because they fear they will be hurt. There are those who flee out of disgust for the mentally ill. There are also others who get close and provide care. There are those who provide care like their own.

I: What kind of care do they provide?

P: From psychology perspective and give cloth, and dress them when they are naked. There are also people who provide psychological support by talking to the person and strengthening by reading books and who help them recover and get on the line.

I: Do members of the community help them?

P: Some support and others do not.

I: Do you know why don’t they support?

P: Because I told you earlier some think he will hurt them, some are disgusted by them, and some are just careless.

I: What does the community think causes mental illness?

P: First it might be caused by addiction, for example one person takes different addictive drugs like cigarette, Hashish, Ganja etc and one day if he doesn’t get the drug on time he will experience mental disorder. It also comes from over stress from work or family, thinking a lot and extreme worry. For example a person who heads a household thinks a lot and when you think too much and your mind can not handle it you may become mentally disordered. It may also occur from witch craft or spirits, magic spells and cult worship.

I: What do you mean by cult worship?

P: There are people who go to witches, who serve the devil, like this. The community also may call somebody by a bad name and make him what he is not.

I: How does the community view a person with mental illness?

P: A person with mental illness is seen more different than a normal person by the community. He may be different in his action or general condition.

I: What do you mean when you see not normal thought or action?

P: For example when he does things that are different or unique from the culture of the area he lives and grow in. when he does that repetitively he may be considered as abnormal.

I: When he does it repetitively?

P: Yes.

I: What do you think should be done by the community and you?

P: I should tell him it is not correct when he does things that are different from the community, I should provide care, I should get close to him and help him return to his consciousness. I should give him my attention like a healthy person and understand and help him.

I: What kind of support do you think will give?

P: That person is mental so he is not normal and I should give him support different from a normal person. If he is walking naked I should cover him up and if I can I should take him to a medical institution, if medication is provided to him I should make sure he takes his medication, and if he has fallen I should pick him up. I should give him different types of support for him.

I: Ok. How does the community view mental health institutions and are there such institution in the area?

P: There is no mental health treatment center for now but if there was taking them there would be good.

I: How are mentally ill people get treatment around here?

P: In the area I live people have strong faiths and they take them to their respective religious institutions the Muslims to a Muslim place, the Orthodox to a holy water and most of them use traditional methods because there is no center for treatment of mental illness.

I: Which one does the community choose between medication and traditional /religious treatment?

P: In my community they choose religious because there is no medical center for treatment around here. They don’t have awareness to go to other places and they don’t have the economic capacity to do so. In our locality they choose religious institutions.

I: Explain care for a mentally ill person with example.

P: If one mentally ill person doesn’t have the medication to take on time, make sure he takes it, if he went to a holy water site then help him to finish his baptism and get cured, and motivating the person by talking with him about ideas that elevate his spirit. To build his mental capacity we should explain more than we do for a normal person. Giving food, cloth, and different support for them is good.

I: How and by who are the care provisions you talked about?

P: It can be provided by any one a man or a woman. There is a thing called humanity for example you see something and you just can’t leave it, they say human is a cure for human. Care giving doesn’t take identity, religion, ethnicity and any one can provide care.

I: Have you provided care and support? If you have share your experience.

P: There are some in my surrounding, once I went to church and one woman fainted, and together we took her to her place. This is the small contribution t I made and I see it in different places but I couldn’t get close and help them.

I: Have you thought you might face metal illness?

P: I didn’t think about now. I am not mentally ill, I am healthy right now. But no one knows what will happen in the future.

I: If you had mental illness who do you think will help you?

P: I think the community will help me, the surrounding community, the community I grow up in is known for supporting each other. The support starts with family and goes from there and I think anyone could help me.

I: What about medical institutions, holy water and things like that?

P: Yes. First the community supports me and other than that they will prepare the situation for me to receive other forms of support. They for example will take me to a medical center or to a holy water.

I: From whom do they prefer to get support and why?

P: I prefer to get help from God first. I am a firm believer and I believe God helps better than people. That is why I prefer the help of God as first.

I: Explain why you prefer that.

P: Because the creator is all above all humans, he is the creator and humans are his creation. I think tht God can help me better than humans and I do truly believe that.

I: From who does the community prefer to get support and why?

P: I think the community should give first place for God like me. People may get bored, may be uncomfortable and God doesn’t want any benefit. God is above humans and we are his creatures so he will help us.

I: Given the current situation what do you think must be done about mental illness starting from the government?

P: The government should build mental illness recovery centers since there isn’t around here. It should provide awareness about the causes of mental illness, if they are youth for example teach them not to begin addiction. The government should aware the use using written material and other media. Other than that there should be a rehabilitation center.

I: What should individuals do?

P: Rather than stigmatizing and being disgusted by a mentally ill person, they should care and take him to a better health setting. If there is a holy water they should take him to there or to a center, they should take it there. The community can also support that person psychologically.

I: Ok. If you think you have an idea that must be covered tell me.

P: I think the government should build a mental illness rehabilitation center in here. They also should aware the youth including causes and solution.

I: OK.

P: The community should provide care rather than stigmatizing and should help in the recovery of that person’s health.

I: I have finished my questions, these were the main ideas I wanted to raise. Thank you for your good participation and cooperation.

P: Thank you for selecting and interviewing me.
