# Supplementary material for: “People are shortening the lifetime of mentally ill persons”; Community’s perception towards mental illness and help-seeking behavior in Bench Sheko, Sheka, Kaffa and West Omo zones, South West Ethiopia, 2021
Source: PLoS One. 2025 Apr 29;20(4):e0320740. doi: 10.1371/journal.pone.0320740 (PMC12040187; doi:10.1371/journal.pone.0320740)
Supplement: S1 File — (ZIP) [file pone.0320740.s001.zip › Transcribed data sample/Interview data (H).docx]

**Research title: *Community Perception and Help-seeking Behavior Towards Mental Illness and Its Associated Factors among Bench-Sheko, Kaffa, West Omo and Sheka Zone***

**Region: SNNPR**

**Interview category**: KII

**Setting:** Rural

**Key:-**

**I:-Interviewer**

**P:-Participant**

I: ok as I told you earlier; our discussion point is about Community perception and help seeking behavior towards mental illness and its associated factors, so please tell me what mental illness means.

P: Mental illness is a disease which is very harmful, which isolates the person from social life. A mentally ill person is unconscious of himself, about the country and other people. Mentally ill people don’t get support and care rather they will be fired from home or their residence, so they move to towns, live on the street and face different harms.

I: What symptoms do these mentally ill people do have?

P: The first thing is loss of consciousness or unable to think as he may do before.

I: Ok was there any one who has experienced mental illness among your family?

P: No, there is no one.

I: How do the community members see mental illness?

P: Our communities don’t hate mental illness as that of epilepsy, they don’t push ill people because they consider as the illness is God’s will.

I: How does the community call the mentally ill people?

P: They call them mad (Lolu), people may tie the mentally ill people and let he stays at one place than giving necessary care or taking to treatment area.

I: What are causes of mental illness as per the community’s thought?

P: Sometimes mental illness is associated with evil spirit (Kalcha), especially in rural area. People thought that if children fail to worship or commit what is needed to their belief in relation to kalcha, as that of their families, they may face mental illness. Again also people don’t speak loudly; stress is also a cause of mental illness. Another evil spirit related issue is the so called likift and muart (sew be lela sew lay yehone neger/metet maserat)

I: What should be done for a mentally ill person? how the community think?

P: There is no tangible support rather people abuse mentally ill people by calling them mad, shouting and laughing on them.

I: Where do people take a mentally ill person for treatment?

P: Orthodox Christians take to holy water and we have witnessed that many people got recovered from mental illness. Others who believe in Kalcha also do something related to that (worship Devil) and sometimes recover. Some go to hospital like to Amanuel Hospital

I: What health facility is there which may support mentally people?

P: There is no health facility around us and I didn’t see when health professionals support them.

I: Where people prefer to take a mentally ill person for treatment?

P: People usually prefer the traditional and religious places; because one; they believe in their faith and the second reason is, people don’t afford to take to hospital.

I: What care does a mentally ill person need? Please tell me examples of cares.

P: Usually people who have strong family get support otherwise most live in the street.

I: Have you ever given care to a mentally ill person?

P: I try to support them in providing cloth, food and even contribute money and send them for health care service.

I: Do you think as you may face mental illness?

P: Why not because I am also a human being..

I: Whom do you think may help you if you face mental illness?

P: God and family.

I: From whom do prefer to get a support or treatment; Modern or traditional or spiritual?

P: Firstly I prefer the religious support.

I: What should be done regarding mental illness from government, NGOs and other stakeholders?

P: Good if everyone contributes its own part. Of course the first one could be the community because they are close to mentally ill people.

I: Do you have any additional points?

P: No.

I: I have finished, Thank you!

P: Ok! Thank you!
