# Supplementary material for: “People are shortening the lifetime of mentally ill persons”; Community’s perception towards mental illness and help-seeking behavior in Bench Sheko, Sheka, Kaffa and West Omo zones, South West Ethiopia, 2021
Source: PLoS One. 2025 Apr 29;20(4):e0320740. doi: 10.1371/journal.pone.0320740 (PMC12040187; doi:10.1371/journal.pone.0320740)
Supplement: S1 File — (ZIP) [file pone.0320740.s001.zip › Transcribed data sample/Interview data (I).docx]

**Research title: *Community Perception and help Help-seeking Behavior towards mental illness and Its Associated Factors among Bench-Sheko, Kaffa, West Omo and Sheka Zone***

**Region- SNNPR**

**Interview category**: KII

**Setting:** Rural

**Key:-**

**I:-Interviewer**

**P:-Participant**

I: Ok as I told you earlier; our discussion point is about Community perception and help seeking behavior towards mental illness and its associated factors, so please tell me what mental illness means.

P: A mentally ill person someone who doesn’t control himself and may do unnecessary things.

I: What symptoms do these mentally ill people do have?

P: Mentally ill people may walk here and there, fails to concentrate, may not dress cloth and may talk alone.

I: Ok was there any one who has experienced mental illness among your family?

P: No, there is no one.

I: How do the community members see mental illness?

P: Mentally ill people are called Mad (Ibid).

I: What are causes of mental illness as per the community’s thought?

P: If head of a newly born child touches the land or if the child fails on the ground and its head touches the ground; we think that it may get mental illness.

I: What is the perception of the community towards mental illness?

P: Families try to take the person to hospital.

I: What should be done for a mentally ill person? how the community think?

P: As much as possible people try to take mentally ill person to hospital.

I: Where do people take a mentally ill person for treatment?

P: People go to traditional healers like to Kalcha

I: Where people prefer to take a mentally ill person for treatment?

P: Formerly people used to prefer to take to traditional places but currently they visit health facilities.

I: Have you ever given care to a mentally ill person?

P: Sometimes I try to support.

I: Do you think as you may face mental illness?

P: I may face but God knows.

I: Whom do you think may help you if you face mental illness?

P: Family.

I: From whom do you prefer to get a support or treatment; Modern or traditional or spiritual?

P: Firstly I prefer health facilities and the community do also.

I: What should be done regarding mental illness by the government, NGOs and other stakeholders?

P: Good if everyone contributes its part and supports mentally ill people.

I: Do you have any additional points?

P: No.

I: I have finished, Thank you!

P: Ok! Thank you!
