# Supplementary material for: “People are shortening the lifetime of mentally ill persons”; Community’s perception towards mental illness and help-seeking behavior in Bench Sheko, Sheka, Kaffa and West Omo zones, South West Ethiopia, 2021
Source: PLoS One. 2025 Apr 29;20(4):e0320740. doi: 10.1371/journal.pone.0320740 (PMC12040187; doi:10.1371/journal.pone.0320740)
Supplement: S1 File — (ZIP) [file pone.0320740.s001.zip › Transcribed data sample/Interview data (J).docx]

**Research title: *Community perception and help seeking behavior towards mental illness and its associated factors among Bench-Sheko, Kaffa, West Omo and Sheka Zone***

**Region-SNNPR**

**Interview category**: In depth interview

**Setting:** Rural

**Transcriber name**:

**Key:-**

**I:-Interviewer**

**P:-Participant**

I: ok as I told you earlier; our discussion point is about Community perception and help seeking behavior towards mental illness and its associated factors, so please tell me what mental illness means.

P: It is not common in our area but I think it results from substance abuse like alcohol intake, chat chewing and also related to business problem.

I: what symptoms these mentally ill people do have?

P: In the beginning it seems as someone drunken. They may shout, put off their cloth and walk bare body. They may collect dirty materials and walk on the street. Stay on the street day and night despite they have home.

I: Ok was there any one who has experienced mental illness among your family?

P: No, there is no one.

I: I addition to what you told me earlier; what other causes do bring mental illness?

P: Sometimes mental illness is associated with evil spirit (Kalcha), especially in rural area. But in urban area there are youth whom I know healthy before 3 or 4 years but now mentally ill because of addiction of chat and cigarate. Again while it could be managed, they lack support from families. In addition, lack of medical service and social support aggravates the situation.

I: Have you ever faced mental illness?

P: No.

I: What should be done for a mentally ill person?

P: People should support mentally ill persons, take them to health facility and health professionals should also give necessary treatment and support.

I: Are there mentally ill people in your area?

P: Yeaha; sometimes they look healthy. But later they may put off their cloth and walk barely.

I: what health facility is there which may support mentally people?

P: there is no health facility and I didn’t see when health professionals support them.

I: Where people prefer to take a mentally ill person for treatment?

P: there is no modern and traditional treatment center, but I think it would be good if both are available.

I: if one of your family members faces a mental illness; where could you take for treatment service?

**0:06:00**

P: If the medical area is well organized, I will prefer it but know I will go to holy water.

I: What care does a mentally ill person need? Please tell me examples of cares.

P: Firstly, it is important to know the reasons behind the illness; we have to know the harming things. Following that; there should be a body which puts solution for that and that body may not be merely governmental body but also social groups or charity organizations.

I: Have you ever given care to a mentally ill person?

P: There is no system which facilitates for it but sometimes I participate in financial support to take a mentally ill people for holy water.

I: Do you think as you may face mental illness?

P: It is something which may occur incidentally so I may face or not.

I: Whom do you think may help you if you face mental illness?

P: It is the public; I don’t think family alone could bring solution.

I: From whom do prefer to get a support or treatment; Modern or traditional or spiritual?

P: Firstly I prefer the spiritual support because we have no accesses to the modern treatment center in our area. But we have holy water nearby and get the aid soon.

I: What should be done regarding mental illness from government, NGOs and other stakeholders?

P: You know many of mentally ill persons could get recovery if they get support. Mental illness usually gets complicated as we undermine and fail to give attention considering as minor thing. So the public should take the lion share and also the government too. It is also necessary to build a facility where such services could be given and to have the right health professional as well.

I: Do you have any additional points?

P: The health sector is responsible for this issue and should take the highest part. Mentally ill people who could heal easily are suffering from lack of necessary treatment, you know there is no treatment and no one is supporting them. So the public and government should cooperate and bring solution; at least good to facilitate for them to reach to Amanuel Hospital.

I: I have finished, Thank you!

P: Ok! Thank you!
